# Evaluation of Anti-nociceptive and Anti-inflammatory Activities of Novel Chalcone Derivatives

**Published:** 2013

**Authors:** Ali Razmi, Afshin Zarghi, Sara Arfaee, Nima Naderi, Mehrdad Faizi

**Affiliations:** a *Department of Pharmacology and Toxicology, School of Pharmacy, Shahid Beheshti University of Medical Sciences, Tehran, Iran.*; b *Pharmacology and Applied Medicine Department of Medicinal Plants Research Center, Institute of Medicinal Plants, ACECR, Karaj,Iran. *; c *Department of Medicinal Chemistry, School of Pharmacy, Shahid Beheshti University of Medical Sciences, Tehran, Iran.*

**Keywords:** Chalcone, Cyclooxygenase, Anti-nociceptive activity, Anti-inflammatory activity, Writhing test, Paw edema test

## Abstract

Chalcone (1,3-diarylprop-2-en-1-one) derivatives have been introduced as selective cyclooxygenase-2 inhibitors. In the present study, anti-nociceptive and anti-inflammatory effects of eight novel compounds were evaluated in male mice and Wistar rats by using the writhing and formalin-induced paw edema tests respectively. The activities of the compounds were compared with celecoxib as a reference drug. Then, novel compounds were divided into two regioisomeric groups based on the position of the methylsulfonyl substitution. Compounds with substituents such as: 1) H, 2) Me, 3) F and 4) Cl at *para *position of the phenyl ring of (E)-3-(4-Methanesulfonylphenyl)-1-phenylprop-2 en-1-one were selected in the first group. The regioisomer compounds with 5) H, 6) Me, 7) F and 8) OMe substitutions at C-4 of phenyl ring of (E)-1-(4-Methanesulfonylphenyl)-3-phenylprop-2-en-1-one were chosen as second group. All compounds showed dose-dependent anti-nociceptive activity in writhing test. Interestingly, the potency of anti-nociceptive effect of compounds 1, 2, 5 and 6 were significantly higher than celecoxib. The regioisomeric compounds 1 and 5 with high anti-nociceptive effects, showed a significant dose-dependent anti inflammatory activity in the paw edema test as well. The results showed that compounds with no substituent or small size substituents at *para *position of the phenyl ring are the most potent compound in writhing test. Our results revealed that the introduction of a bulky group such as methoxy or chlorine at the vicinal aromatic chain of the derivatives decreases the anti-inflammatory/ anti-nociceptive effects. The comparison of estimated ED_50_ of each pair of the regioisomeric compounds indicates that the relative position of SO_2_Me to carbonyl moiety did not affect the potency.

## Introduction

There are couples of mediators involving in the inflammation process including cytokines, cell adhesion molecules and autacoids such as prostaglandins (PGs). PGs, especially PGE_2_, increase the tissue blood flow and cause pain (1, 2). Non-steroidal anti-inflammatory drugs (NSAIDs) are the inhibitors of prostaglandin G/H synthase (cyclooxygenase; COX) and are used in attenuation of pain and inflammation. Unwanted effects on different systems, such as gastric mucosa and coagulation process, are related to the reduction of PG synthesis due to the inhibition of COX enzymes (3). Discovery of COX-2 isoform, an isoform predominantly expressed in inflamed tissues, unlike the COX-1 isoform which is constitutively expressed with homeostatic functions, encouraged researchers to synthesize selective COX-2 inhibitors (4, 5). The inhibition of COX-1 is the main cause of gastric ulcer, a significant side effect of NSAIDs. COX-2 enzyme has a negligible role in gastrointestinal homeostasis, but it may assist COX-1 in protecting gastric mucosa against damaging stimuli (6, 7). Clinical trial studies revealed that COX-2 selective inhibitors are more tolerable than traditional NSAIDs due to the diminished gastric side effects (8-10). Despite some debates about the cardiovascular safety of COX-2 selective inhibitors (11) which led to the withdrawal of rofecoxib from market, further studies did not find any relationship between the COX-2 selective inhibition and the increase in risk of cardiovascular problems in compare with the traditional NSAIDs (12).

 NSAIDs, except for salicylate derivatives, block arachidonic acid access to tyrosine 385 at the apex of catalytic channel of COX-1 and COX-2. There are some sequential differences between two isoforms of COX which have been utilized by medicinal chemists to produce selective COX-2 inhibitors. A crucial difference between the two isoforms of COX is at the position of 523 of the enzyme where valine is substituted for isoleucine. This makes a side pocket on the COX-2 enzymatic active site which is missed in COX-1. In addition, the existence of a large aromatic amino acid (phenylalanine) at the position 503 in COX-1 prevents the access of COX-2 selective inhibitors to the active site of COX-1,while the small leucine at the same position in COX-2 provides an extended space for the action of large COX-2inhibitormoiety (13).

One of the frequently used structures to obtain a selective COX-2 inhibitor is the substitution of a pharmacophore group such as SO_2_NH_2_ or SO_2_Me at the *para *position on diarylheterocycles class. In concordance, *para*methylsulfonylchalcone (1,3-diarylprop-2-en 1-one) derivatives showing COX-2 selective inhibitory properties in molecular modeling and *in-vitro *studies were made (14). This study is designed for *in-vivo *evaluation of the antinociceptive and anti-inflammatory effect of different chalcone derivatives which can lead to the better understanding of the structure-activity relationship in COX-2 selective inhibitors and will give information about the anti-nociceptive and anti-inflammatory efficacy of these novel compounds.

## Experimental

*Animals*

Male NMRI albino mice weighing 18-22 g and male Wistar rats weighing 150-250 g were used. All animals accessed to the standard food and water and were housed in 12-h dark/light cycle in a controlled condition at 23-25ºC. All animals were transferred to the experiment room 2 h before the trial. This study is approved by the institutional animal care and use committee of Shahid Beheshti University of Medical Sciences and all pharmacological experiments were performed in accordance with National Institute of Health (NIH) Principles of Laboratory Animal Care (NIH publication #85-23). In all tests, adequate considerations were adopted to reduce pain or discomfort of animals.

*Compounds*

**Figure 1:**
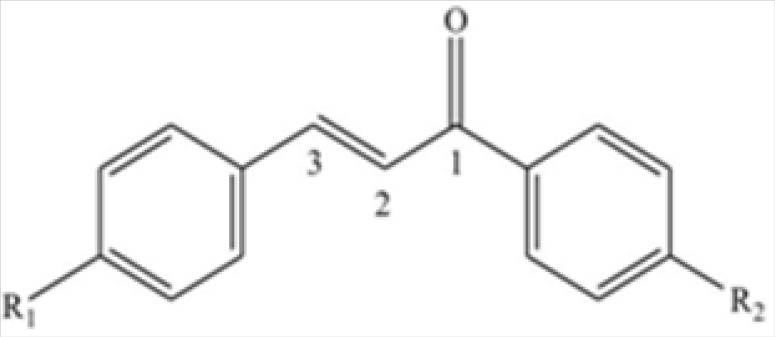
Chemical structure of the chalcone derivatives

Celecoxib powder (Sigma-Aldrich), formalin 5% made from formaldehyde 37% and acetic acid (Merck, Germany) were used. The novel chalcone derivatives were synthesized based on the previously described methods (14). The novel compounds were divided into two regioisomeric sets. In the first group, methylsulfonyl was placed at the *para *position of the phenyl ring attached to the C-3 of chalcone and H, Me, F, and Cl^-^ substitutes were positioned at *para* position of the phenyl ring attached to the C-1 of the derivatives (1, 2, 3 and 4 respectively). In the second set H, Me, F, and OMe substitutes were placed at the *para *positions of C-3 phenyl ring of chalcone-derived substances (5, 6, 7 and 8 in the same order), and pharmacophore moiety (methylsulfonyl) was placed at the *para *position of the C^-1^-conjugated phenyl ring (see Figure 1). Selectivity and potency of these compounds were previously shown by ligand binding assay (14). Celecoxib and chalcone derivatives were dispersed in a solution containing 1% of Tween 80 in normal saline.

Group1: R_1_ = SO_2_Me; R_2_: 1 = H, 2 = Me, 3 = F, 4 = Cl

Group2: R_2_ = SO_2_Me; R_1_: 5 = H, 6 = Me, 7 = F, 8 = OMe

*Writhing test*

Mice were used for the writhing test to study the anti-nociceptive activities of the novel compounds. The study was performed at 0900 h to 1300 h. Animals were divided into groups of eight each. Tween 1% in normal saline was used as vehicle (control group). Celecoxib and the novel compounds were dispersed in the vehicle by shaking and using ultrasonic machine, and were administered via intraperitoneal (4) injection. Thirty minutes later, 0.1 mL acetic acid 1% was injected peritoneally. The response was assessed by counting the number of abdominal constrictions (writhes) for the duration of 21 min, starting 5 min after the injection of acetic acid solution (15, 16). The dose of the compounds which could reduce the number of writhes to 50% of the mean value of the control group was considered as estimated ED50. Each animal received only one dose of any drug and participated only in one trial.

*Formalin-induced paw edema test*

The anti-inflammatory activities of celecoxib and the novel compounds were assessed using the rat paw edema test utilizing 40 μL formalin 5% as a phlogistic agent. A mark was made on the lateral malleolus and the paw was dipped in mercury column under the sign and the volume of paw was calculated as described before (17, 18). The initial volume of paw was measured before the injection of formalin solution. Then, the novel compounds, celecoxib, or Tween 1% saline, as the control solution, were administrated intraperitoneally. After 45 min, formalin was injected subcutaneously into the subplantar tissue of the right paw of all groups. The volume of paw was measured in different treatment groups, 4 h following the formalin injection. The amount of increase in paw volume (edema volume) was calculated by subtracting the volumes before and 4 h after the injection of formalin (19). The dose which could reduce the edema volume to 50% of the mean value of the control group was considered as estimated ED50 of celecoxib, and the novel compounds as well. Each animal received only one dose of each drug and participated only in one trial.

*Statistical analysis*

Data were reported as mean ± SEM. Estimated ED50 and exact confidence intervals were determined using the method described by Tallarida (20). The slop deviation of the doseresponse curve was analyzed using Fieller’s theorem. All mentioned computations were performed by using Microsoft Excel. One-way ANOVA followed by Tukey’s post test was used to compare applied doses of each compound with control group. P < 0.05 was considere significant in all tests.

## Results

The results of anti-nociceptive effects of compounds are shown in Table 1. All compounds showed a significant analgesic effect in writhing test. Compounds with hydrogen or methyl substituent had more potency among the first group of the compounds which have methylsulfonyl group at *para *position of the first phenyl (conjugated with C=O group directly). Assortment of potency of the compounds in the first group was as follows: 1 = 2 > 4 > 3. In addition, the ED50 of compounds 1 and 2 were significantly less than that of celecoxib. Amongst the compounds of the second group, with methylsulfonyl substituted at *para *position of C-3 phenyl, hydrogen or methyl substitution showed the maximum potency which was significantly more than the reference drug celecoxib. Potency arrangement in this group was: 6 **> **5 **> **8. Although significant reduction in writhes was observed in mice treated with compound 7 at the doses of 10, 20 and 40 mg/ kg compared to control group, a significant dose-response relationship was not determined for this compound. There was no significant difference between the two regioisomeric groups.

**Table 1:** Effects of 8 chalcone-derived compounds on acetic acid induced abdominal constriction in mice; Response represents the number of constriction; *p < 0.05, **p < 0.01, ***p < 0.005 significant differences compared to control group (n = 8); p-values: significance level for deviation of slope from zero; ND: Not determined due to no significant dose-response relationship.

**Compound**	**R1**	**R2**	**Dose (mg/kg)**	**Response (n) Mean ± SEM**	**Estimated ED50 Mean (95%Confidence Interval) p-value**	**p-value**
Control			0	34.4 ± 5.8		
**Celecoxib**			10	23.7 ± 6.0	0.0339	0.0339
			20	18.5 ± 2.9		
			40	12.5 ± 4.0 *		
			80	10.6 ± 5.2 *		
*1*	SO2Me	H	1.25	21.5 ± 3.3	2.2 (1.3-3.7)	0.0000
			2.5	20.0 ± 3.9		
			5	6.1 ± 3.1 ***		
			20	2.6 ± 1.46 ***		
*2*	SO2Me	Me	5	17.2 ± 4.7	3.2 (0.9-10.3)	0.0076
			10	5.8 ± 1.7 ***		
			20	7.1 ± 3.1 ***		
			40	3.1 ± 1.4 ***		
*3*	SO2Me	F	20	36.2 ± 8.7	61.8 (38.1-99.9)	0.0085
			40	17.3 ± 6.4		
			80	17.2 ± 5.7		
			100	9.7 ± 3.9		
*4*	SO2Me	Cl	5	25.8 ± 3.8	16.7 (9.9-27. 9)	0.0025
			10	22.1 ± 3.6		
			20	17.5 ± 4.5		
			40	8.7 ± 4.2 **		
*5*	H	SO2Me	5	19.6 ± 4.9	8.1 (4.0-16.5)	0.0265
			10	18.3 ± 3.9		
			20	6.1 ± 2.0 **		
			40	8.0 ± 2.8 **		
*6*	Me	SO2Me	1.25	15.1 ± 4.1	1.1 (0.3-2.9)	0.0073
			2.5	15.6 ± 2.5		
			5	7.1 ± 1.7 **		
			10	5.6 ± 2.5 ***		
*7*	F	SO2Me	2.5	12.7 ± 3.2	ND	0.1601
			5	16.6 ± 7.5		
			10	12.8 ± 3.9 *		
			20	11.5 ± 3.1 *		
			40	8.5 ± 3.0 **		
*8*	OMe	SO2Me	20	30.1 ± 6.2	61.8 (36.5-104.6)	0.0037
			80	13.7 ± 3.2		
			160	6.8 ± 5.1 **		

The results of the paw edema test are reported in Table 2. 

**Table 2:** Effects of 8 chalcone-derived compounds on formalin induced paw edema in rat. Response represents the reduction of volume of inflamed paw; *p < 0.05, ** p < 0.01 significant difference compared with control group (n = 8); p-values: significance level for deviation of slope from zero; ND: Not determined due to no significant dose-response relationship

**Compound**	**R1**	**R2**	**Dose (mg/kg)**	**Response (n) Mean ± SEM**	**Estimated ED50 Mean (95%Confidence Interval) p-value**	**p-value**
Control			0	0.221 ± 0.018		
**Celecoxib**			10	0.218 ± 0.021	65.9 (29.2-148.5)	0.0078
			20	0.186 ± 0.027		
			40	0.088 ± 0.024 **		
			80	0.154 ± 0.019		
*1*	SO2Me	H	5	0.220 ± 0.015	26.2 (13.8-50.0)	0.0020
			10	0.148 ± 0.014 *		
			20	0.137 ± 0.015 *		
*2*	SO2Me	Me	5	0.170 ± 0.008	ND	0.0836
			10	0.156 ± 0.025		
			20	0.148 ± 0.014		
			40	0.128 ± 0.016 **		
*3*	SO2Me	F	10	0.204 ± 0.026	ND	0.1622
			20	0.210 ± 0.008		
			40	00.232 ± 0.052		
			100	0.246 ± 0.008		
*4*	SO2Me	Cl	20	0.254 ± 0.039	ND	0.5036
			40	0.218 ± 0.007		
			80	0.229 ± 0.023		
*5*	H	SO2Me	2.5	0.209 ± 0.013	40.4 (11.8-137.6)	0.0029
			5	0.164 ± 0.018		
			10	0.136 ± 0.010 *		
			20	0.144 ± 0.013 **		
*6*	Me	SO2Me	5	0.185 ± 0.021	ND	0.0840
			10	0.164 ± 0.019		
			20	0.175 ± 0.022		
			40	0.185 ± 0.026		
			80	0.112 ± 0.012 **		
*7*	F	SO2Me	2.5	0.216 ± 0.026	37.7 (6.9-204.4)	0.0339
			5	0.169 ± 0.032		
			10	0.164 ± 0.018		
			20	0.134 ± 0.024		
*8*	OMe	SO2Me	20	0.199 ± 0.028	ND	0.5036
			40	0.156 ± 0.026		
			80	0.133 ± 0.024		

Among the compounds of the first group, compound 1 (10 and 20 mg/kg) and compound 2 (40 mg/kg) showed significant anti-inflammatory effects compared to the control group. However, compounds 3 and 4 did not show a significant anti inflammatory effect in the administered doses. The anti-inflammatory properties of compound 1 were comparable with celecoxib (p > 0.05). In group 2, Compounds **5 **(10 and 20 mg/kg) and 6 (80 mg/kg) showed a significant anti-inflammatory effect compared to the control group. Compound 7 and 8 did not produce any significant anti-inflammatory effect in the administered doses.

However, a significant net effect in reducing paw edema (p = 0.034) was observed for compound

7. The anti-inflammatory effect of compounds 5 and 7 were comparable with celecoxib (p > 0.05). Moreover, compounds 1 and 5**, **which are regioisomers, showed no significantly different effect in the test.

## Discussion

A group of novel chalcone derivatives possessing a methyl sulfonyl group at the *para* position of the C-3 phenyl ring having different substituents (H, Me, F, and Cl; 1-4) and the corresponding regioisomers (H, Me, F, and OMe; 5-8) were synthesized previously to investigate the effect of these substituents on anti-nociceptive and anti-inflammatory activities. The *in-vitro* COX-2 inhibitory studies of these compounds showed that the type of the *para-*substituents and the position of the SO2Me pharmacophore on the C-1 or C-3 phenyl ring are important for their activities (14). According to our results, the compounds with no substitution or small groups such as methyl at *para *position of C-3 (1 and 2) or C-1 (5 and 6) phenyl ring are the most potent anti-nociceptive agents in both groups. Interestingly, the potency of these compounds is even higher than reference drug celecoxib. In contrast, the introduction of a larger group such as Chlorine or Methoxyphenyl (4 and 8) decreases the anti-nociceptive activity.

These might be explained by steric parameters. On the other hand, compounds having strong electron withdrawing group such as F (3 and 7) show lower activity. These findings indicate that the nature and the size of substituents are important in anti-nociceptive effects of the novel compounds. These results are in agreement with the previous *in-vitro *study (14).

Compounds with no substitution at *para *position of C-3 or C-1 phenyl (1 and 5) have anti-inflammatory activity comparable with celecoxib. Although the regioisomers with methyl ubstitution (2 and 6) have no significant anti-inflammatory dose-response relationship (p = 0.08 for both regioisomers), at higher doses they show a significant difference in reduction of paw edema compared to vehicle. Since compounds with Cl and OMe substitutions did not show significant anti-inflammatory effects, it could be concluded that bulky substitutions reduce the anti-inflammatory activity of this group of compounds. In parallel with the results of anti-nociceptive evaluation, the regioisomers with F substitution having no anti inflammatory activity suggest that the presence of an electron-withdrawing moiety significantly reduces the anti-inflammatory effect of the compounds. The position of methylsulfonyl at C-1 or C-3 phenyl ring does not affect the anti-nociceptive and anti-inflammatory effects of this series of the compounds. It could be explained with the similar orientation of the regioisomers in interaction with COX-2. The results also indicate that the antinociceptive potency of the novel chalcone derivatives is higher than that of their anti-inflammatory effect. This may be related to other mechanisms of chalcone derivatives involving in pain control (21).

Our results revealed that the methylsulfonyl chalcone-derived compounds can be potent analgesic and anti-inflammatory compounds, if the proper substitutes bind with each phenyl ring at the *para *position. Further pharmacological and toxicological experiments could be led to novel development of drugs for managing pain and inflammation. 
